# Addressing multimorbidity to improve healthcare and economic sustainability

**DOI:** 10.15256/joc.2016.6.74

**Published:** 2016-02-17

**Authors:** Francesca Colombo, Manuel García-Goñi, Christoph Schwierz

**Affiliations:** ^1^Health Division, Organisation for Economic Co-operation and Development (OECD), Paris, France; ^2^Department of Applied Economics II, Universidad Complutense de Madrid, Madrid, Spain; ^3^Directorate General, Economic and Financial Affairs (DG ECFIN), European Commission, Brussels, Belgium

**Keywords:** Multimorbidity, multiple chronic conditions, comorbidity, Organisation for Economic Co-operation and Development, integrated care, health economics, finance, resources

## Abstract

Patients with multimorbidity are responsible for more than half of all healthcare utilization, challenging the healthcare budgets of all European nations. Although the European Union is showing signs of a fragile economic recovery, achieving sustainable growth will depend on delivering a combination of fiscal responsibility, structural reforms, and improved efficiency. Addressing the challenges of multimorbidity and providing more effective, affordable, and sustainable care, has climbed the political agenda at a global, European, and national level. Current healthcare systems are poorly adapted to cope with the challenges of patients with multimorbidity. Little is known about the epidemiology and natural history of multimorbidity; the evidence base is weak; clinical guidelines are not always relevant to this population; and financing and delivery systems have not evolved to adequately measure and reward quality and performance. Pockets of innovation are, however, beginning to emerge. In Spain, for example, the ongoing economic crisis has forced regional governments to deliver substantial efficiency savings and, with this in mind, integrated care programmes have been introduced across the country for people with chronic disease and multimorbidity. Early results suggest that formalized integrated care for patients with multimorbidity improves their perceptions of care coordination, reduces hospital and emergency admissions and readmissions, and reduces average costs per capita. Such innovations require meaningful investments at a national level – something that is now supported within the framework of the European Union’s Stability and Growth Pact.

## Introduction

The burden of disease has changed dramatically in recent decades, and chronic conditions are now the most common reason people seek medical help. It has been estimated that 85% of all healthcare utilization is by people with at least one chronic condition, and 65% is absorbed by people with multimorbidity [1]. The European economy, while turning a corner, remains fragile, with very high levels of public debt and healthcare budgets still under strain. Along with technological advancements, our ageing populations and increasing incidence of chronic disease and multimorbidities are putting unprecedented pressure on healthcare systems – pressure that will only intensify if changes are not made. Making healthcare more sustainable is now an urgent priority within Europe and tackling multimorbidity in a more effective, affordable, and sustainable way has climbed the political agenda. In this review article, three influential economists and policymakers review where we are today in terms of developing more sustainable health systems to tackle multimorbidity and what may still be required at a European and national level to reduce the burden of disease.

## Reforming health systems to meet the challenges of multimorbidity

Population ageing continues to increase demands on health and long-term care systems and, in today’s economic climate, the challenge is to preserve access to high-quality care at an affordable and sustainable cost. Even taking into account the slowdown in health spending following the economic and financial crisis, healthcare costs are likely, according to projections, to keep on rising in advanced economies, so that, without major reform, they will be unaffordable by the middle of the century [2]. Much of the upward pressure on healthcare spending relates to new technologies, but the growing needs of ageing populations and the substantial costs associated with managing large numbers of people with chronic conditions and multimorbidity play an important part [2].

In 2011, recognizing the need for health system reform to address the challenge of patients with multimorbidity, the Organisation for Economic Co-operation and Development (OECD) published a report, “Health reform: meeting the challenge of ageing and multiple morbidities” [3]. The messages from this report are still very real today. There are very many areas in which our health systems are poorly adapted to cope with patients with multimorbidity, and although some progress has been made, significant improvements are still needed.

### Biomedical research

Significant overall gains in population health have been achieved across OECD countries in the past decade [4]; however, our ability to predict the health and care needs linked to multimorbidity remains poor. Biomedical and health service research is fundamental to changing the way healthcare is delivered, generating the evidence base on which decisions are made. Unfortunately, research on chronic conditions typically focuses on tightly defined single entities (e.g. diabetes, asthma, hypertension), while overlooking the impact of multimorbidity and frailty on health and other outcomes. Clinical trials of new drugs or other treatments often prospectively exclude older individuals or patients with comorbidity, leading to a scientific evidence base that does not apply to most individuals presenting in clinical practice. This, in turn, leads to the development of clinical practice guidelines that are not necessarily relevant to the patient with multimorbidity. If the evidence base is not generated for patients with multiple chronic conditions, it is difficult to design quality metrics and payment and delivery systems that are relevant and evidence based. When considering reforming health systems to meet the challenges of multimorbidity, investing in biomedical research that improves our understanding of its natural history and how best to manage it would ultimately enable us to develop more sustainable methods to deliver care.

### Medical education/training

Another key issue relates to the relevance of the traditional medical curriculum to the management of individuals with multimorbidity [3]. Medical education has traditionally focused on single-disease systems – a focus that is still largely in place today. Patients with multimorbidity have multiple care needs that are best coordinated and addressed in primary care. Unfortunately, primary care is a relatively underdeveloped aspect of healthcare in many countries, with primary care training typically shorter than that of specialist training, and education relating to the care of the elderly, chronic disease management, and ambulatory care coordination limited. Since there is good evidence that countries with strong primary healthcare systems have better health outcomes [5, 6] and that living in a country with a robust primary care system benefits patients with multimorbidity [7], investing in primary care education and strengthening primary care systems seems an obvious solution to delivering more sustainable care.

### Financing and delivery systems

Options for financing health and social care for individuals with multimorbidity remains a subject of intense debate today. In 2011, the OECD report described us as being “at a crossroads” in terms of how we plan future financing and delivery systems to meet the challenges of multimorbidity, with two very different options proposed [3]. The first option described a pathway that led to bundled payments, transferred risk, and traditional market competition. Along this pathway, systems would be developed that involved the creation of standardized and widely disseminated care plans for a wide range of medical conditions, with the measurement of performance based on a range of clearly defined outcomes. Along the alternative pathway, locally based, whole-system targets, pooled budgets, and innovative market models would be developed that were value- not process-driven and evolved from within. 

The past 4 years have witnessed a shift in the way we think about designing and financing healthcare systems, and although consistent progress in this direction has been slow, glimpses of innovation have been observed. A recently reported study identified several innovative financing methods that have been used in Europe to improve the delivery of integrated care for people with multimorbidity, with pay-for-performance approaches rewarding the quality of care provided [8]. In Japan, healthcare providers have been rewarded/remunerated for specific activities targeted towards care coordination for people with multimorbidity, with fees applied if, for example, care plans are in place or lifestyle advice has been given [9]. Other innovative models involving payments following clinical pathways; population-based payments; and pay-for-performance schemes are being implemented across several OECD countries [10]. All too often, however, traditional financing models are still used in the management of patients with multimorbidity, which do not incentivize providers to coordinate or integrate care or to manage these patients in a more holistic or efficient way. Considerably more work is needed in this important area of health service reform.

### Quality metrics

Finally, the issue of quality metrics in multimorbidity care must be addressed more fully, as experience in the development and use of such indicators is limited. Most existing quality indicators are focused on individual medical conditions, and studies examining quality of care for multiple conditions simply combine these individual indicators into a single outcome. Qualitative studies of people with multimorbidity suggest that care integration, coordination, and continuity are all critical facets of quality care [11], yet these have all been identified as problems entrenched within current healthcare systems around the world [12]. 

Several quality indicators appear to be valid, feasible, and useful to policymakers addressing multimorbidity: firstly, coordination and planning of care for individuals should be measured to assess patients’ experiences of discontinuity, duplication of care, and health service errors. These could be measured in simple metrics, as whether or not the individual has a named main provider of care or a named coordinator of care, what information is recorded in the patient’s notes, or what measures have been taken to address polypharmacy. Secondly, individual-level measures of the effectiveness and safety of care should be incorporated, including the incidence and severity of adverse events and what actions have been taken to protect the safety of the individual. Finally, patient- and carer-reported outcomes, such as quality of life, activities of daily living, and patient experiences, should be considered as key quality indicators when assessing multimorbidity care.

### The right direction

In summary, we have made some progress in recent years at a local, national, and international level in the way our health services are evolving to better meet the needs of patients with multimorbidity. We still have a long way to go before we are delivering sustainable care of acceptable quality, but at least we are now travelling in the right direction.

## Improving efficiency through integrated care for patients with multimorbidity: the Spanish experience

The ongoing economic crisis in Spain has led to unprecedented levels of unemployment and dramatic reductions in gross domestic product (GDP) per capita, with poverty rising at an alarming rate. This has had a major impact on funding and provision for the healthcare system. While financing through taxes in the Spanish National Health System is centralized, healthcare planning and provision is largely decentralized and managed by autonomous regional governments who are responsible for its costs. When the central government in Spain mandated that regional public expenditures should be reduced, policymakers found themselves urgently seeking efficiency savings, while still trying to guarantee universal coverage, equitable access, and quality of care.

**Figure 1:**
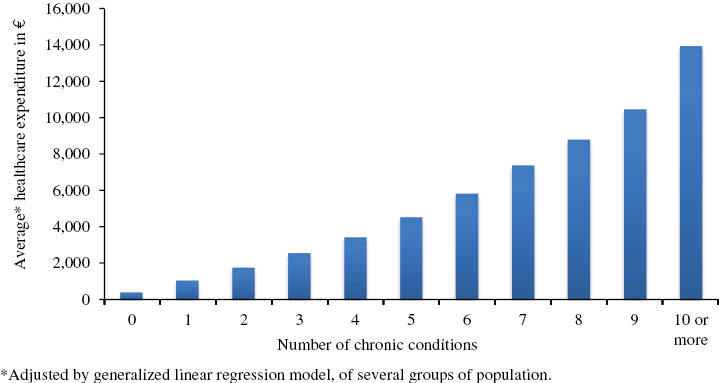
Healthcare expenditure associated with multimorbidity in a population-based study in the Basque Country of Spain [13]. Adapted from Orueta JF, *et al.* Prevalence and costs of multimorbidity by deprivation levels in the Basque Country: a population based study using health administrative databases. PLoS One 2014;9:e89787. CC BY-SA.

The management of chronic diseases, in general, and multimorbidity, in particular, absorbs a substantial proportion of healthcare budgets in Spain. A recent study conducted in the Basque Country – a region in northern Spain with a population of approximately 2.2 million – found that 24% of the population had multimorbidity, which present in 66% of those aged over 65 years [13]. The prevalence of multimorbidity was higher in women than in men, and highest in the most deprived areas in the region. The annual cost of healthcare increased in a non-linear manner according to the number of chronic conditions present, with a mean cost of €1,017 for the first condition, rising to €13,891 when 10 or more conditions coexisted [13] (Figure 1). 

What this and other studies have illustrated is that healthcare expenditure is concentrated in patients with multimorbidity and that more efficient management of these patients should lead to improved outcomes and cost savings. The need to have a strategy in place to address the challenges associated with long-term conditions was recognized many years ago in Spain, and the first “Strategy to tackle the challenge of chronicity” was launched in the Basque Country in 2010 [14], with other regions following suit. The Ministry of Health, Social Services, and Equality also published in 2012 its strategy to improve the quality of healthcare provision to patients with chronic conditions for the entire Spanish National Health System [15]. Today, all regions in Spain have a strategy for chronic care in place, encouraging collaboration between healthcare providers and the development of local systems offering continuity of care.

The Spanish experience has demonstrated that it is possible to transform healthcare delivery focusing on chronic care if that transformation is system-wide, and based on primary care support, widespread innovation, and investment in information technology [16]. Integrated and effectively coordinated care is pivotal to the chronic care models developed in Spain, and many different programmes are underway to deliver more integrated care for people with long-term conditions and multimorbidity. In the Basque country alone, 11 Integrate Care Organizations are now operating, and it is anticipated that, within the next 3 years, integrated care will be available to the entire population of the region [17].

Early experiences from the integrated care programmes underway in the Basque country have been encouraging [17]. One of the first programmes to deliver results was steered by the Bidasoa Integrated Health Organization, which was the first integrated healthcare organization launched in the Basque Country and one of the first in Spain, with the vision to build a new organizational model for care of patients with chronic conditions based on culture, clinical practice, and governance [17]. A more patient-orientated approach to collaborative practices was developed and the first Continuity of Care Unit was created to treat patients with complex long-term health needs. Organizational performance has been assessed using Spanish adaptations of the Assessment of Readiness for Chronicity in Healthcare Organizations (ARCHO), the D’Amour questionnaire and a Triple Aim-based dashboard. Patient experiences of care, hospital utilization, and per capita costs have also been evaluated. Overall improvements have been reported in terms of the organizational readiness for chronicity and in collaborations between professionals at different care levels [17]. Patients’ perceptions of care coordination have improved, hospital admissions and readmissions have decreased, especially within the group of patients with complex or multiple conditions, and the average per capita costs have reduced, although this cannot necessarily be attributed directly to the provision of more integrated care [17]. 

Implementation of strategies to address the pathway towards a chronic care-focused healthcare system is one of the main concerns in health policymaking in Spain [17]. Although these are relatively early days, further analysis of other ongoing programmes, given by the high degree of decentralization, will enhance the evidence base and may lead to additional improvements in the way we deliver care to patients with multimorbidity and complex health needs. In the next phase of reforms, it is hoped that health and social care can be brought closer together to work in a more integrated way, i.e. an improved way that would particularly benefit patients with multimorbidity.

## Taking a Europe-wide view: how the European Commission is tackling efficiency and sustainability

The European Union (EU) has at last turned a financial corner, with economic growth slowly returning. There are promising signs of a rebalancing in the EU economy and significant efforts are being directed towards achieving sustainable economic growth, higher levels of employment, and financial stability. The mission of the European Commission’s Directorate-General (DG) for Economic and Financial Affairs (DG ECFIN) is to contribute to raising the economic welfare of EU citizens by developing and promoting policies that ensure sustainable economic growth [18]. In order to achieve this, DG ECFIN focuses on three key areas: fiscal responsibility, structural reforms, and efficiency. 

Public debt has soared in the last 5 years, and by the end of 2014, debt levels across the Eurozone had reached an all-time high of almost 92% of GDP, with 16 countries struggling with debts larger than the 60% of GDP limit set out in the Maastricht Treaty. The Stability and Growth Pact, which was introduced to strengthen the monitoring and coordination of national fiscal and economic policies in order to reduce public debt and protect the Euro, has recently been re-evaluated [19]. The revised Stability and Growth Pact makes room for member states to invest in health by strengthening the link between structural reforms, investment, and fiscal responsibility in support of jobs and growth [19]. The Commission takes into account the positive fiscal impact of structural reforms on growth and the long-term sustainability of public finances in the so-called “structural reform clause”. Also, public investments under the Pact are easier, and under certain conditions, member states can deviate from their fiscal objectives in order to accommodate investment, e.g. on health-related projects co-funded by the EU under the Structural and Cohesion Policy. This bodes well for future health sector reforms, with a more flexible approach allowing for meaningful investments in healthcare by member states.

The Commission has recognized in the staff working document, “Investing in health”, that besides being a value in itself, health is also a precondition for economic prosperity [20]. The healthcare sector has major economic significance: it represents 10% of the EU’s GDP. The healthcare workforce accounts for roughly 8% of all jobs in the EU. The 2015 Annual Growth Survey, which kicks off the European Semester of European economic governance, makes clear that efficiency and financial sustainability of healthcare systems needs to be improved, increasing their ability to meet social needs and ensure essential social safety nets. It acknowledges the importance of the healthcare sector in tackling the social consequences of the economic crisis, stressing significant job opportunities in the health sector in the years to come. As part of the European Semester process, which sets out country-specific recommendations for policy reforms in EU member states, in 2015, 11 EU countries have received a recommendation for reforming their health systems. Most of these recommendations focus on the cost-effectiveness of health systems, calling for reforms in the hospital sector, outpatient care, and primary care, with some explicitly calling for maintenance of or improvement in the access to and quality of healthcare. The country-specific recommendations ultimately aim to improve the value-for-money for public and private payers of healthcare services, knowing that in all EU countries there are possible shortcomings and potential areas for improvement, notably in terms of lower costs (savings) and improved cost-effectiveness (better health with same costs) in the health sector. This integrated approach should ultimately benefit the EU population. 

Health and long-term care spending, in particular, poses a major fiscal challenge across Europe, with most member states facing issues relating to the sustainability of public finances in the medium- and/or long-term. It has been estimated that most of the increase in age-related expenditure between 2013 and 2060 will be due to healthcare and long-term care, with public spending projected to increase by 2–4% of GDP in that time [21]. These are conservative estimates and do not account for the issue of rising levels of multimorbidity, simply because the data do not exist at a European level to enable us to project health expenditure in this area. The Third EU Health Programme 2014–2020 recognizes the challenges posed by the increasing prevalence of chronic disease [22] and points to innovation and increased efficiency to achieve more sustainable health systems. Efficiency is an issue the European Commission takes very seriously, with major work underway to improve the efficiency of current healthcare systems across all EU countries [23]. Results obtained to date suggest that, on average in the EU, life expectancy at birth could be increased by 2.3%, or 1.8 years, if healthcare systems became more efficient [23].

The question of how to improve healthcare efficiency across Europe is not an easy one to answer, and requires a set of actions and a collaborative approach at the European level. The recent Commission communication, “On effective, accessible and resilient health systems”, sets the agenda for various Commission actions, which strengthen the effectiveness of health systems, increase the accessibility of healthcare, and improve the resilience of health systems [24]. The Commission set up the Expert Panel on Effective Ways of Investing in Health in July 2013. The panel develops recommendations focusing on primary and hospital care, pharmaceuticals, research and development, health economics, and eHealth, among others. The Commission develops expertise on the performance assessments of health systems, collaborating with member states and key international health organizations, helping member states to draw the best lessons possible from recent health system reform experience and to show where performance can be improved. 

The issue of multimorbidity clearly needs to move up the European agenda, with better data available to assist policymakers and health service planners. A centralized resource would help to capture the experiences and learnings from the many different projects underway around Europe aimed at improving multimorbidity care efficiency, enabling member states to exchange ideas and disseminate best practice. 

The European Commission is committed to supporting future European fiscal and growth and to enabling its member states to deliver efficient and sustainable healthcare. How we better integrate the growing challenge of multimorbidity in our policy agenda remains to be determined.

## Summary and conclusions

The high costs associated with managing multimorbidity in an increasingly ageing population are putting unsustainable pressure on the fragile European economy. Innovative strategies are required to deliver high-quality healthcare at an affordable and sustainable cost, with health systems being reformed at every level to meet the challenge. At a European level, tackling multimorbidity will require a greater understanding of the extent of the problem across the region, with health and social care policymakers working alongside economists to identify current inefficiencies and optimize future planning. Nationally, a clear focus on integrated care and care continuity for people with multimorbidity has the potential to increase both the quality and cost-effectiveness of care, and the results of ongoing integrated care programmes are awaited with interest. In developing more sustainable models of care in multimorbidity, a key goal must be to improve our understanding of its epidemiology and natural history, and the best ways to both prevent and manage it, to strengthen primary care, and to encourage the development of innovative financing and delivery systems that measure and reward quality and performance.
