# Erratum to: Addressing multimorbidity to improve healthcare and economic sustainability

**DOI:** 10.15256/joc.2016.6.77

**Published:** 2016-03-01

**Authors:** Francesca Colombo, Manuel García-Goñi, Christoph Schwierz

**Affiliations:** ^1^Health Division, Organisation for Economic Co-operation and Development (OECD), Paris, France; ^2^Department of Applied Economics II, Universidad Complutense de Madrid, Madrid, Spain; ^3^Directorate General, Economic and Financial Affairs (DG ECFIN), European Commission, Brussels, Belgium

**Erratum to:** Colombo F, García-Goñi M, Schwierz C. Addressing multimorbidity to improve healthcare and economic sustainability. J Comorbidity 2016;6(1):21–27. doi: 10.15256/joc.2016.6.74.

The first sentence of the Acknowledgements section should read, ‘The opinions expressed in this paper are the responsibility of the authors and do not necessarily reflect those of the OECD, the European Commission, or its member countries.’

Journal of Comorbidity 2016;6(1):33

The original article can be found at: http://dx.doi.org/10.15256/joc.2016.6.74

